# Mozart's Requiem–Liver Transplantation in 1988

**DOI:** 10.1155/1990/69704

**Published:** 1990

**Authors:** J. M. Little

**Affiliations:** Department of Surgery Westmead Hospital Westmead N.S.W. 2145 Australia

## Abstract

Liver transplantation is one of the most spectacular of surgical achievements. It is a demanding and expensive procedure, requiring great surgical skill and a great depth of supporting services. Precisely because it is a procedure at the leading edge of medicine, more and more units in developed countries are pressing to be allowed to carry it out. But there are many moral and ethical problems, some of which can be usefully examined using a “Mozart model” as proposed by Starzl.


*HPB Surgery*, 1990, Vol. 2, pp. 15–20
Reprints available directly from the publisher
Photocopying permitted by license only

© 1990 Harwood Academic Publishers GmbH
Printed in the United Kingdom

### MOZART'S REQUIEM — LIVER TRANSPLANTATION IN 1988

J. M. LITTLE

*Professor of Surgery, Department of Surgery, Westmead Hospital, Westmead*

*(Received 10 April 1989)*

Liver transplantation is one of the most spectacular of surgical achievements. It is a demanding and expensive procedure, requiring great surgical skill and a great depth of supporting services. Precisely because it is a procedure at the leading edge of medicine, more and more units in developed countries are pressing to be allowed to carry it out. But there are many moral and ethical problems, some of which can be usefully examined using a "Mozart model" as proposed by Starzl.

KEY WORDS: Liver transplantation, cost benefit.

I had the privilege of attending a meeting of a surgical society devoted to the study of diseases of the liver, biliary tree and pancreas in Bologna during the autumn of 1988 — a privilege partly because it was one of the meetings held to celebrate the nine hundredth anniversary of the University of Bologna. It was a good meeting which focused much of its scientific attention on the medical problems of liver transplantation. Indeed, one of its central purposes was to honour Dr Thomas Starzl of Pittsburgh for his astonishing achievements as a pioneer of liver transplantation. Some of the Italian speakers also reported their small and early experiences, while Bismuth from Paris, Broelsch from Chicago and Otte from Louvain in Belgium reported the experience of busy and well established units in other countries.

This was stirring material indeed. All the speakers emphasized that the operation saves lives and can produce good quality of life in the survivors. Furthermore, the study of the biology and immunology of liver transplantation can produce new insights into the function of the liver in health and disease. The success of liver transplantation can produce new insights into the function of the liver in health and disease. The success of liver transplantation had, moreover, had significant social impact. It was dramatic and demanding of resources. If transplantation was not available, the patient was usually doomed since there is no hepatic equivalent of a dialysis machine which will artificially duplicate the multitudinous functions of the liver. For these reasons, more countries had confronted the problem of defining brain death to allow optimal conditions for harvesting the liver; and more countries were coming to terms with the concept of self-recycling, allowing such measures as endorsement of the driving licences of potential donors. Unfortunately, for various financial, religious and social reasons, these benefits and changes had not evolved in China, Africa, India nor the Islamic countries. While the developments were striking and remarkable, they were available to a good

Requests for reprints: Professor J. M. Little, Department of Surgery, Westmead Hospital, Westmead 2145 N.S.W. Australia.

deal less than half the world's population. Despite this, liver transplantation has done much to nurture international exchange. Students from many countries were spending time at the established transplant units, and organs were being sent across national boundaries.

Starzl addressed delegates twice — once in a formal lecture, once on receiving the honorary Laurea of the University of Bologna. He has some interesting and striking messages. He pointed to the high success rate now achieved by liver transplantation, with 70% of a population with no other future alive at one year. He felt some doubts about the propriety of transplanting patients with active hepatitis B virus infection and those with most malignant tumours of the liver. Neither of these groups did well, because the diseases which prompted the transplant recurred either in the new liver or elsewhere in the body. On the other hand, he had found that alcoholics had fared remarkably well, and he thought that reform from drinking was made easier by the very drama and stress of the operation. It is certainly remarkable to hear that a group of alcoholics have reformed so completely. Experience of other conditions, such as bleeding varices and chronic pancreatitis, is much less favourable.

Starzl also pointed out that liver transplantation has allowed some biological access to problems such as hypercholesterolaemia, and that experience of a single case could shed more light than years of work in a laboratory. In short, he saw transplantation generally and liver transplantation in particular as one of the keys to modern medicine and its relationship to society. He asked his audience at the presentation of his honorary Laurea to consider the history of music if transplantation had been available for Wolfgang Amadeus Mozart. To have added years to Mozart's life might have added music of sublime quality. Mozart's contemporary musical influence might have spread. The history of music might have been very different.

Amidst this euphoria, there was one small discord. Otte, talking of liver transplantation in children, warned his audience that the cost of providing a paediatric transplant service for European countries outside Belgium was becoming too great in Louvain, and that some limits would need to be set. This warning was directed particularly to his Italian hosts, who supplied most of the children of whom Otte was speaking. To an outsider, this was a puzzling reference to cost, which was not mentioned elsewhere in the lectures and presentations.

At the lunch table, however, there was a different agenda. There was concern about the availability of livers, and concern about the regulation of access to available organs. Methods of supply and demand are regulated largely by finance. At one centre in the U.S.A., the asking price for a liver transplant is $170,000, at another $135,000. State funding for the programme in Louvain can barely cope with the needs of children from other countries. In Italy, state funding has meant the imposition of quotas for each of the funded transplant units. One major service, for example, is allowed to do only 20 transplants each year.

Some remained doubtful about the advisability of transplantation in alcoholics, feeling that patient selection was all important. An alcoholic with enough money to pay for a transplant is likely to be better motivated than one who lives on a state pension. Most were anxious that Starzl's message could be taken too literally, without a careful analysis of the type of alcoholic being seen in each country by the units performing transplant surgery.

But most concern was shown over the nexus between finance and the availability of the operation. It is worthwhile to examine this issue further. There seem to be four socio-economic models to be considered — the free-market, the fully regulated, the mixed and the open systems.

Under the free-market system — as seen to some degree in the United States — the user pays, and if the user cannot pay he or she does not have a liver transplant. The justifications of this system are easily made. By hiving off this expense to the private sector, the government is not directly involved and does not perceive the high cost as a major threat to its health budget. At least some people have access to a method of treatment that saves lives. The economically successful and their families deserve this access. The problems are just as easily identified. The personal cost is enormous. If the cost of the operation is to be covered by insurance schemes, insurance premiums will rise, the more so as the number of operations rises. The wealthy benefit not only from exclusive availability of the operation. Liver transplantation uses resources, including large amounts of blood, which are not then readily available to charity patients. Nor is it fair to say that only the rich deserve the operation. There are worthwhile members of the poor. Mozart was one of them. Finally, if the wealthy are the only group to benefit, decisions that should be made on medical grounds will be made for economic reasons.

Is the fully regulated system — as seen in Italy — a better one? In this model, government funds the programmes, and — at least in theory — the operation is available to all for medical, not economic indications. But since government supplies the funds, the budget is controlled by government which must also find money for other areas of health, defence, education, the arts and roads. Such a system demands careful definition of guidelines for the inclusion and exclusion of patients with liver disease. There does not seem to have been a concerted attempt to define these criteria, and indeed it .may not be possible to develop anything more than the broadest guidelines. If a limit of 20 cases in each year is imposed on a particular unit — as it is for one Italian unit — , then a few emergency transplants will completely destroy a carefully planned waiting list. In France, the audience was told by Bismuth, a donor liver will be available within 24 hours when an emergency is notified to the national transplant agency. In other countries, availability is less certain. What is certain is the confusion caused by the insertion of an emergency case at the top of the planned waiting list.

The mixed system allows a government regulated stream to run alongside a privately funded stream. This may seem at first to be more equitable, but it is unlikely to be so. Government, perceiving that private enterprise is taking a share of the financial burden, is likely to reduce the funding for the public sector. One country will therefore have an 'open' stream for the wealthy and a 'restricted' sector for the poor. Equity is unlikely to flourish for long.

A completely open system is one in which all citizens have equal access to transplantation. Inclusion in the programme and priority on the list are determined by medical need alone. The expense is a national one, to be met from taxes and insurance premiums. While this may be seen to be an ideal system, it may only be possible in egalitarian countries like Sweden and Australia. Further, it may only be possible in countries with small populations, since a large population will generate such a pressure of numbers that the starting budget will soon blow out, with a predictable response from government. It will obviously be possible only in countries that are relatively wealthy and that have at least some leaning toward a welfare state. It is also likely that programmes of this kind will develop in countries that are relatively unimportant in world politics. In the more important, the defence budget will compete too much. Sadly, the very success of such a system will contain the seeds of its own destruction. As its reputation grow, the pressure on its facilities will increase. As more personnel are hired and more equipment bought, costs will spiral. Eventually government will intervene, and an open system will be converted into a fully regulated one.

It is interesting to consider the fate of Mozart as a potential transplant recipient under these different systems. But first it is necessary to digress a little and to consider the nature of Mozart's earlier health and his final illness[1,2]. Despite his abundant vitality, Mozart's health was never robust. As a child, he probably suffered from recurrent streptococcal infections, with erythema nodosum, rheumatic fever, Henoch-Schonlein purpura and perhaps glomerulonephritis. He also endured typhoid, smallpox, dental abscesses, frostbite, recurrent bronchitis and an episode of jaundice (presumably caused by hepatitis A virus).

His final illness was called "acute military fever" on his death certificate, but this non-specific diagnosis, referring to fever and skin rash, does not help us much. We know from contemporary accounts that for some weeks before his death Mozart had been unwell and depressed. He had suffered from several blackouts and he complained of joint pains. He developed generalised oedema, fevers and vomiting. His son mentioned a terrible smell that filled the sick room. The presence of a rash can be inferred from the diagnosis of acute military fever. Two hours before death, Mozart suffered from convulsions, became unconscious and was noted to turn his head to one side and to breathe with his cheeks puffing out.

Dr P.J. Davies[3] has postulated that Mozart suffered from longstanding immune complex disease caused by recurrent streptococcal infection. He probably suffered from chronic renal failure for some years. His final illness was perhaps precipitated by a further bout of streptococcal infection contracted at a Masonic Lodge meeting on 18th November, 1791. There is evidence that many people contracted a similar infection during a particularly severe winter in Vienna. The immune complex disease that followed caused acute renal failure with hypertension and Henoch-Schonlein purpura. The uncontrolled hypertension and low platelet count ultimately produced a cerebral haemorrhage.

This line of speculation may or may not be correct. Starzl's point was that a transplant — presumably renal — might have saved Mozart's life. So, he could have added, might better hygiene, control of hypertension, antibiotics, steroids and renal dialysis. The possibilities are so many that the exercise becomes almost pointless. But there is some point in considering Mozart as a potential transplant candidate, particularly if we extend the fantasy and assume that he needed a liver transplant. Liver transplantation is more expensive and less readily available than renal transplantation.

Let us first look at his background. He was loved by many musicians — like Haydn and J.C. Bach — and by many of his contemporaries. But he was not particularly favoured by those in power, amongst whom he had influential acquaintances but few friends. He had suffered innumerable rebuffs and slights from the wealthy and powerful in Salzburg, Mannheim, Munich, Prague and Paris. Only during his terminal illness was he granted his first tenured post as kapellmeister at St Stephen's Cathedral in Vienna. Until then, he had relied on commissions and the sale of his works. He was, at the time of his death, desperately poor. The equivalent of about $200 in contemporary terms was found about the house. An inventory of his effects revealed possessions to the value of about $2000. His debts totalled about $3000. Over the preceding few years, he had repeatedly borrowed from his brother Masons. Prosperity might have been around the corner, but it had not arrived.

Under which system would Mozart have got his hypothetical liver transplant? Under the open market system? He certainly could not have afforded it personally. Wealthy sponsors were not in evidence, having shown little tendency to help before his death. Under a private system, a transplant would have cost about 50,000 gulden of

1791, which would have been a lot for the Masons to find, particularly as the Masons were being restricted in their activities and freedoms by Leopold II in Vienna. In the fully regulated and mixed systems, Mozart would enter the hepatic transplantation list for the poor on medical grounds, and no one could be too sure how his history of repeated previous infections would have affected his chances of inclusion on the list. If he was entered during his final, fulminant illness (all over in about 2 weeks), he might have got his transplant in France, but presumably only if he were a French national. If he were to be transplanted during the chronic phase, he would have had to wait his turn in his own country, and wait for a suitable donor. If a "quota" of transplants had been done for the year, he might not have been transplanted at all. Only under an open system could he have been guaranteed equitable access to the transplant list.

Even this brief and incomplete analysis of the "Mozart problem" shows how dangerous it is to use historical analogies out of all context. The unknowns are too many, the certainties too few, — and this without even touching on the possible effects of immune suppressive regimes on creativity. But the examination of the case of Mozart has been useful in clarifying some of the ethical issues, which must persist even in the most starry-eyed view of hepatic transplantation. It is not available to the bulk of the world's population in China, India and Africa, countries in which liver disease is particularly common. It is most available where wealth is most available. Details of the costs and benefits under the different schemes of health care delivery are not clear. And no one seems keen to investigate the numbers at risk globally and to determine what facilities should be made available worldwide. Nor does anyone seem keen to confront the issues of wealth as the first pre-requisite in a free market system, of arbitrary limits in the regulated and mixed systems and of the eventual self-destruction of the open system.

There is, in short, a need to think through some of the issues that surround liver transplantation. First, the profession must examine its attitude to concentrating expertise. Liver transplantation is so demanding of technical expertise and resources, both human and physical, that its performance must be confined to a few units only. The trouble is that once a procedure is deemed to be at the cutting edge of medical endeavour, more and more units feel it necessary to provide that procedure. We saw how destructive that could be to cardiac surgery in the last generation. The profession will have to confront this issue now. Hepatic transplantation is too expensive to be allowed to proliferate in the traditional way until each city has 8 units performing 20 transplants each year, instead of one major unit performing 160, with concentration of expertise and economy of scale.

Second, though no less important, the profession and the politicians must determine a way to make hepatic transplantation equally available to the rich and the poor. This statement has nothing to do with personal political views. It is a statement of belief in a fundamental human right. Even the most rabid free-marketeer will agree that some are poor because of illness. They must have the right to be made whole. (This applies only to developed countries. It does not even touch on the moral dilemma that liver transplantation raises by widening the gap between the developed and undeveloped countries.)

Third, each society must determine what proportion of national wealth can be set aside for hepatic transplantation. On this decision will turn the size of quotas in the regulated system, to which I suspect all systems will converge in time.

Fourth, the profession must work hard and honestly to define the indications for and the contra-indications to transplantation. The issues of self-destruction by alcohol, of viral persistence, of tumour recurrence must all be studied and reviewed — but not by everyone at once. We do not have models of all these things which can be readily taken to the laboratory. Our only model is the illness of man. Let each unit try to solve the problems that most beset them and that they are best equipped to solve. If all the transplant units try to solve the problem of transplantation for hepatitis B infection, many wasted transplants will be performed. If one or two units push forward such experimental work with virologists who have new therapies available, then we may have progress with the greatest economy of human life and medical resources. Above all things, the transplanters must communicate with one another. They are working with a most expensive and precious experimental model. Their work must be co-ordinated. They will need to be more generous to one another than is usual among the medical profession.

There are many other issues to be confronted at both national and international gatherings. They include the balance between coercive law and national choice that must be made by each community, and the effects that such decisions may have on the international exchange of donor organs. Will a country that introduces some laws to procure more donor organs feel obliged to send organs to other countries that leave organ donation to the choice of its people? And what should be done about those countries that feel religious, moral or social reluctance to donate, although perhaps no such reluctance to receive?

Ultimately, Starzl is right to see liver transplantation as a key to modern society. It is unfortunately also a key to a Pandora's box. In that box are issues that will test the sincerity of both governments and the medical profession. They will test to the limit a government's real commitment to individual health; and they will equally test to the limit the profession's ability to concede and concentrate expertise.

We could do worse than to propose a simple test for clinicians and administrators to apply to their established or proposed hepatic transplant units. It might be called The Mozart Test. It consists in answering honestly the question "Is this unit available for certain to provide a transplant for a sick, impoverished, powerless man, who may or may not be a genius?" If the answer is "Yes", then the unit will have fulfilled at least its first obligation.

*References*

1. Levey, M. The life and death of Mozart. Sphere Books Ltd, 1988
2. Davies, P.J. Mozart's illnesses and death. Musical Times, 1984 CXXV;437–461, 554–61
3. Robbins Landon, H.C. Mozart's last year. Thames and Hudson, London, 1988

*Accepted by S. Bengmark 15 April 1989*

